# Risk assessment instruments for pressure ulcer in adults in critical situation: a scoping review

**DOI:** 10.1590/1518-8345.6659.3984

**Published:** 2023-10-06

**Authors:** Ricardo Jorge de Barros Romeira Picoito, Sara Maria May Pereira da Cruz Lapuente, Alexandra Catarina Parreira Ramos, Isabel Cristina Mascarenhas Rabiais, Sérgio Joaquim Deodato, Elisabete Maria Garcia Teles Nunes

**Affiliations:** 1 Universidade Católica Portuguesa, Escola de Enfermagem do Instituto de Ciências de Saúde, Lisboa, Portugal.; 2 Hospital de São Francisco Xavier, Centro Hospitalar Lisboa Ocidental, Lisboa, Portugal.; 3 Escola Superior de Enfermagem de Lisboa, Centro de Investigação, Inovação e Desenvolvimento em Enfermagem de Lisboa (CIDNUR), Lisboa, Portugal.

**Keywords:** Risk Assessment, Pressure Ulcer, Intensive Care Units, Predictive Value of Tests, Sensibility and Specificity, Adult, Medición de Riesgo, Úlcera por Presión, Unidades de Cuidados Intensivos, Valor Predictivo de las Pruebas, Sensibilidad y Especificidad, Adulto, Medição de Risco, Lesão por Pressão, Unidades de Terapia Intensiva, Valor Preditivo dos Testes, Sensibilidade e Especificidade, Adulto

## Abstract

**Objective:**

to map the instruments for risk assessment of pressure ulcers in adults in critical situation in intensive care units; identify performance indicators of the instrument, and the appreciation of users regarding the instruments’ use/limitations.

**Method:**

a scoping review. We used the Preferred Reporting Items for Systematic Reviews and Meta-Analyses Extension for Scoping Reviews in the writing of the study. We carried out the searches in the EBSCOhost search tool for 8 databases, resulting in 1846 studies, of which 22 studies compose the sample.

**Results:**

we identified two big instrument groups: generalist [Braden, Braden (ALB), Emina, Norton-MI, RAPS, and Waterlow]; and specific (CALCULATE, Cubbin & Jackson, EVARUCI, RAPS-ICU, Song & Choi, Suriaidi and Sanada, and COMHON index). Regarding the predictive value, EVARUCI and CALCULATE presented better results for performance indicators. Concerning appreciation/limitations indicated by users, we highlight the CALCULATE scale, followed by EVARUCI and RAPS-ICU, although they still need future adjustments.

**Conclusion:**

the mapping of the literature showed that the evidence is sufficient to indicate one or more instruments for the risk assessment of pressure ulcers for adults in critical situation in intensive care units.

## Abstract (es)

**Objetivo:**

mapear los instrumentos para la evaluación del riesgo de lesiones por presión en adultos en situación crítica en una unidad de terapia intensiva; identificar los indicadores de desempeño de los instrumentos y la apreciación de los usuarios con respecto al uso/limitaciones de los instrumentos.

**Método:**

*scoping review*. Para redactar el estudio se utilizó la extensión *Preferred Reporting Items for Systematic Reviews and Meta-Analyses Extension for Scoping Reviews*. La investigación se realizó mediante la herramienta de búsqueda EBSCO*host* en 8 bases de datos, resultando 1846 estudios, de los cuales 22 conforman la muestra.

**Resultados:**

se identificaron dos grandes grupos de instrumentos: los generalistas [Braden, Braden (ALB), Emina, Norton-MI, RAPS y Waterlow]; y los específicos (CALCULATE, Cubbin & Jackson, EVARUCI, RAPS-ICU, Song & Choi, Suriaidi y Sanada y el índice COMHON). En cuanto al valor predictivo, EVARUCI y CALCULATE mostraron los mejores resultados de indicadores de desempeño. En cuanto a las apreciaciones/limitaciones señaladas por los usuarios, destaca la escala CALCULATE, seguida de la EVARUCI y la RAPS-ICU, aunque aún necesitan ajustes futuros.

**Conclusión:**

el mapeo mostró que las evidencias son suficientes para indicar uno o más instrumentos para la evaluación del riesgo de lesiones por presión en adultos críticos en una unidad de cuidados intensivos.

## Abstract (pt)

**Objetivo:**

mapear os instrumentos para avaliação do risco de lesões por pressão nos adultos em situação crítica em unidade de cuidados intensivos; identificar os indicadores de desempenho dos instrumentos e a apreciação dos utilizadores quanto ao uso/às limitações dos instrumentos.

**Método:**

*scoping review*. O *Preferred Reporting Items for Systematic Reviews and Meta-Analyses Extension for Scoping Reviews* foi utilizado para a redação do estudo. A pesquisa foi realizada na ferramenta de busca EBSCO*host* em oito bases de dados, resultando em 1846 estudos, dos quais 22 compõem a amostra.

**Resultados:**

identificaram-se dois grandes grupos de instrumentos: os genéricos [Braden, Braden (ALB), Emina, Norton-MI, RAPS e Waterlow]; e os específicos (CALCULATE, Cubbin & Jackson, EVARUCI, RAPS-ICU, Song & Choi, Suriaidi e Sanada e o índice de COMHON). Quanto ao valor preditivo, a EVARUCI e a CALCULATE apresentaram os melhores resultados de indicadores de desempenho. Em relação à apreciação/às limitações apontadas pelos utilizadores, destacam-se a escala CALCULATE, seguindo-se da EVARUCI e da RAPS-ICU, embora ainda necessitem de ajustes futuros.

**Conclusão:**

o mapeamento mostrou que as evidências são suficientes para indicar um ou mais instrumentos para avaliação do risco de lesões por pressão nos adultos em situação crítica em unidade de cuidados intensivos.

**Highlights:**

**(1)** The risk assessment instrument must be applied to the patient’s specificities.

**(2)** The instruments are divided into two groups: generalist and specific.

**(3)** The EVARUCI and CALCULATE instruments presented better results.

**(4)** The EVARACI presented better results in terms of performance indicators.

**(5)** The CALCULATE highlights itself for being recent scale, appropriate, simple, and easy to use.

## Introduction

Pressure ulcers (PU) constitute a problem that follows health care throughout the time due to the damage it causes to the patient and the costs of the treatment. It is one of the main challenges faced by organizational managers due to the high rate of morbidity, risk of hospital infection, increase in recovery time, and compromise of the patient’s quality of life. It also embraces an increase in the nursing team for the care provision and high costs with specific products for injury treatments^(^ 1 ). PU is considered an adverse event because it is an injury that can be prevented, constituting an indicator of nursing care quality^(^ 2 ).

We define PU as localized damage in the skin or underlying soft tissues, generally over a bony prominence as a result of intense pressure and/or prolonged combined with shear or related to the use of a medical device or an artifact^(^ 3 ).

In Portugal, the prevalence of PU in the hospital environment presents values of 17.4% in hospital services, 7.1% in Surgery, 15.3% in Urgency, and 16.6% in the Intensive Care Unit (ICU)^(^ 4 ). On a worldly level, in ICUs, the prevalence of PU varies from 1.54% to 32.7%, and the incidence from 5.2% to 53.4%^(^ 5 ). In ICU, in particular, there is a higher rate of incidence and prevalence in comparison to other areas of the hospital related to patients in critical situation^(^ 6 ). We understand as patients in critical situation whose life is threatened due to the failure of one or more vital functions and whose survival depends on advanced means of vigilance, monitoring, and therapy^(^ 7 ).

Having as a premise the prevention of PU, it is crucial the identification of the patient at risk, resorting to PU risk assessment instruments. Adult patients in critical situation, hospitalized in ICU, present a multiplicity of risk factors thus, the risk assessment instruments for PU must be specific for the population and context^(^ 8 ^-^ 9 ).

In international literature, there are more than 40 instruments available for the PU risk assessment, although evidence is scarce in suggesting that one instrument is superior to another. However, usually, we consider that the incorporation of an instrument in the formal process of evaluation will help professionals to plan the intervention in PU prevention^(^ 10 ^-^ 11 ).

In general, for the risk assessment of PU development in all care contexts, a generalist instrument is applied that does not consider specific aspects of the patient’s clinical condition. Hence, in risk evaluations of patients in critical situation in the ICU, risk factors of PU development are not contemplated.

A specific assessment instrument for patients in critical situation hospitalized in ICU must take into consideration the peculiarities of their clinical condition, which could enhance accuracy and precision, predicting the risk more correctly^(^ 12 ). In the case of adults in critical situation hospitalized in the ICU, the Braden scale classifies the almost totality of high-risk patients, resulting in many cases of false positives^(^ 12 ^-^ 13 ). This generalized classification hinders the allocation of material and human resources to PU prevention.

The performance indicators that are commonly utilized and recommended in international literature are sensibility, specificity, positive predictive value (PPV), negative predictive value (NPV), and Area Under the Curve (AUC) from Receiver Operating Characteristic (ROC)^(^ 14 ). The sensibility represents the proportion of patients that develop PU evaluated as risky. The specificity regards the proportion of patients that did not develop PU, and the assessment indicated they were not at risk^(^ 15 ). The PPV consists of the proportion of evaluated patients as risky and that, in fact, developed PU. The NPV applies to the proportion of patients that after the assessment are declared as not being at risk and that in fact did not develop PU^(^ 15 ). Another component utilized to compare the predictive capacity of the scales is the ROC curve and more concretely the AUC that “is associated with the discriminant power of a model”^(^ 16 ). AUC values ≤0,5 do not have discriminant power; values between 0.5-0.7 present weak discrimination; between 0.7-0.8, acceptable discrimination; 0.8-0.9, good, and values ≥0.9 exceptional^(^ 17 ).

Scientific evidence suggests that the nursing team, through training and specific knowledge, has an important role in the PU problem. The capacitation of the nursing team allows for assessing the risk correctly, utilizing the most suitable instrument, and implementing preventive actions for skin injuries^(^ 18 ). Many evaluation instruments utilized are, in the majority, selected based on literature and opinions/appreciation of experts^(^ 19 ).

Preliminary research in the search platforms Joanna Brigs Institute (JBI) Evidence Synthesis, Cochrane Database of Systematic Reviews, Cumulative Index to Nursing and Allied Health Literature (CINAHL), PubMed, and Evidence for Policy and Practice Information was developed, and literature review studies were not found, in the development phase or finished, regarding the PU risk assessment instruments in adults in critical situation hospitalized in ICU. It only identified articles that demonstrated the effectiveness of strategies for PU prevention in ICU^(^ 20 ), and articles that showed PU risk assessment instruments previously from 2009 however, directed to the population and hospital context in general^(^ 21 ), and not for patients in critical situation hospitalized in ICU.

Choosing this scoping review derives from the absence of a current literature review work directed to instruments for assessing PU for patients in critical situation hospitalized in the ICU. Therefore, we hope that this study may identify a specific instrument that provides more reliable and trustworthy data about its predictive capacity, considering that risk evaluation is generally carried out through a generic instrument that does not consider the specificities of the patient in critical situation hospitalized in ICU.

The aim of this scoping review consists of mapping the instruments for risk assessment of PU in adults in critical situation in the ICU, identifying performance indicators of the instruments, and the appreciation of users regarding the instruments’ use/limitation.

## Method

### Type of study

We elaborated the present scoping review to allow a broader approach, which aims at mapping the instruments for risk assessment of PU in adults in critical situation in the ICU and provide a general vision of available evidence^(^ 22 ). It is a review that followed the steps recommended by JBI^(^ 23 ), utilizing the Preferred Reporting Items for Systematic Reviews and Meta-Analyses Extension for Scoping Reviews (PRISMA-ScR) for the writing of the study^(^ 24 ). For this scoping review, we did not register the protocol.

### Study scenario

We carried out this review in Lisbon, Portugal, in the database: CINAHL Complete via EBSCOhost, MEDLINE Complete via EBSCOhost, Nursing & Allied Health Collection: Comprehensive, Cochrane Central Register of Controlled Trials, Cochrane Database of Systematic Reviews, Cochrane Methodology Register, MedicLatina, Cochrane Clinical Answers, having as a resource the validated descriptors through CINAHL Subject Headings, MEDLINE – MeSH and Nursing & Allied Health Collection: Comprehensive Subjects and keywords.

### Period

We carried out the timeline of the search of studies from 2008 to April 2023, once the last review found that addressed this theme dated 2007.

### Population

This scoping review analyzed studies about instruments for risk assessment of PU with adult patients hospitalized in the ICU, independently of their pathology or cause of hospitalization, and considered in critical situation. The study population was composed of 1846 scientific articles found in the search carried out in the database and grey literature available on Google Scholar.

### Selection criteria

We included articles with different types of research that approached the instruments for risk assessment of PU for adults in critical situation, hospitalized in the ICU, in English, Portuguese, and Spanish languages, once they are the languages that the researchers are proficient in, from 2008 to April 2023. For the exclusion of studies, we adopted the criteria of being an editor’s letter, abstracts from annals of events, and not presenting information that contemplated the population, concept, and context of interest of this study.

### Study variables

The variables of the study were: title of the article; year of publication; country; journal; language; objective; type of study; performance indicators of the instruments (sensibility, specificity, PPV, NPV, and AUC), and user appreciation regarding use/limitations of the instruments. We carried out the studies through three independent reviewers that evaluated and selected through the title, considering the defined criteria, indexed terms of the subject, abstract, and, when justifiable, we carried out full reading. After data extraction, we resolved the differences that appeared among reviewers through discussion until it reached a consensus.

### Instruments used to information collection

We registered the data extracted from the studies in a data collection tool adapted from a form recommended by JBI, organized in a calculus paper in Microsoft Excel 2016^(^ 25 ).

### Data collection

For the elaboration of the research question, we utilized the mnemonic designated PCC: Participants, Concept, Context. Participants: adults in critical situation. Concept: instruments of PU risk assessment, performance indicators of the instruments, users’ appreciation regarding use/limitations of instruments. Context: hospitalization in ICU, independently of specialty (general, medical, surgical, traumatology, among others, and professional that applied the instrument).

Thus, the research question adopted was “What is the scientific evidence available about instruments of PU risk evaluation in adult patients in critical situation hospitalized in ICU?” Through the research question, we submitted the descriptors and keywords to the crossing of each other, utilizing as a strategy the advanced research form in the aforementioned database.

We included published articles, reviews, and other documents considered relevant to the study, and we carried out the research in three distinct steps: in the first one, a floating research in the database CINAHL and MEDLINE via EBSCOhost was carried out, which we analyzed the articles by the words contained in title, abstract and indexed terms utilized. After this analysis, it was possible to identify the keywords that represent the subject to study and, through them, identify descriptors. In the second phase, we carried out new research according to Figure 1.

**Figura 1 -:** Search strategy conducted in the databases. Lisbon, Portugal, 2023

Database	Search strategy (April 2023)
MEDLINE	“Scales” OR “instruments” OR “Clinical Assessment Tools” AND (MH “Risk Assessment”) OR (MH “Probability”) AND (MH “Pressure Ulcer”) OR (MH “Wounds and Injuries”) AND (MH “Critical Illness”) OR “Critically Ill Patients” AND (MH “Critical Care”) OR (MH “Intensive Care Units”) AND (MH “Sensitivity and Specificity”) OR (MH “Predictive Value of Tests”) OR “Instruments Validation”
CINAHL	(MH “Scales”) OR “instruments” OR (MH “Clinical Assessment Tools”) AND (MH “Risk Assessment”) OR (MH “Probability”) AND (MH “Pressure Ulcer”) OR (MH “Wounds and Injuries”) AND (MH “Critical Illness”) OR (MH “Critically Ill Patients”) AND (MH “Critical Care”) OR (MH “Intensive Care Units”) AND (MH “Sensitivity and Specificity”) OR (MH “Predictive Value of Tests”) OR (MH “Instrument Validation”)
Nursing & Allied Health Collection: Comprehensive	“Scales” OR DE “TEST validity” OR “Clinical Assessment Tools” AND DE “RISK assessment” OR “Probability” AND DE “PRESSURE ulcers” OR DE “WOUNDS & injuries” AND DE “CATASTROPHIC illness” OR DE “CRITICALLY ill” AND DE “CRITICAL care medicine” OR DE “INTENSIVE care units” AND DE “SENSITIVITY & specificity (Statistics)” OR DE “PREDICTIVE tests” OR DE “TEST validity”
Cochrane Central Register of Controlled Trials, Cochrane Database of Systematic Reviews, Cochrane Methodology Register, MedicLatina, Cochrane Clinical Answers	“Scales” OR “instruments” OR “Clinical Assessment Tools” AND “Risk Assessment” OR MH “Probability” AND “Pressure Ulcer” OR “Wounds and Injuries” OR “Decubitus Ulcer” OR “Bed Sore” AND “Critical Illness” OR “Critically Ill Patients” AND “Critical Care” OR “Intensive Care Units” AND “Sensitivity and Specificity” OR “Predictive Value of Tests” OR “Instrument Validation” OR “Predictive Validity”

To complement this step, we carried out another search through additional sources (Google Scholar), which added one study. In the third step, we analyzed the bibliographical references of the selected studies, and did not include new studies.

### Data extraction

We presented the data extracted in the table format organized in chronological order, in which we inserted information about the year of publication, title of the article, publishing journal, country of origin, and study design. The papers added were those that complied with the inclusion criteria, being the research carried out in an international database, selecting primary research articles, systematic reviews, meta-analyses, and academic reports. We did not detect any conflicting interests among authors.

### Data treatment and analysis

We analyzed the articles according to the objectives of the review through content analysis. We present the results in figure format, in which we expose relevant data in compliance with the aim of the scoping review and, later, the narrative descriptive text.

### Ethical aspects

During the development of this study, we complied with the identification of authors using scientific support, as well as carrying out their references as in doing justice to intellectual property.

## Results

**Figura 2 -:**
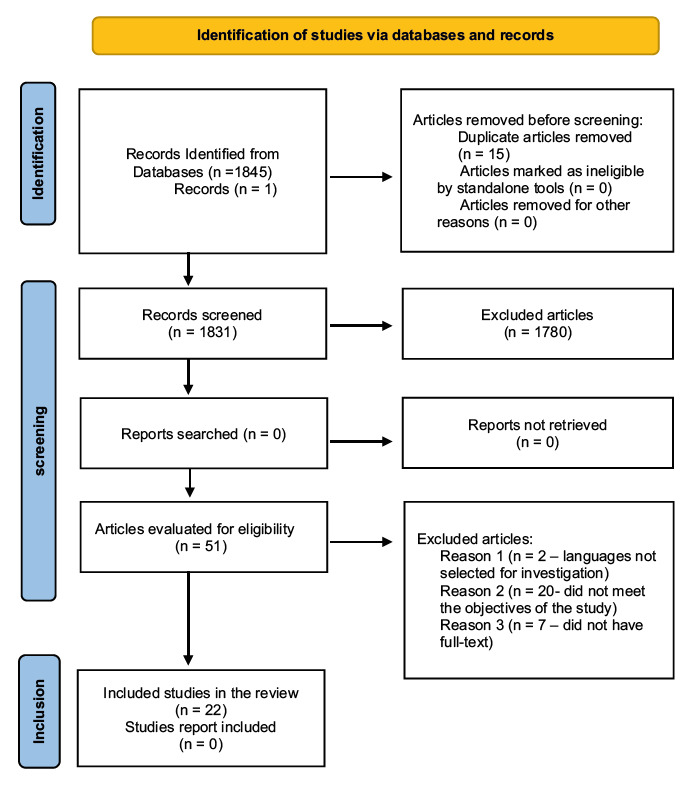
Flowchart of the study selection process adapted from PRISMA-ScR^(^ 24 ). Lisbon, Portugal, 2023

According to an electronic search, we identified in the databases 1846 potentially eligible studies, with 15 articles being removed for being repeated, 1780 after reading the title, terms of the indexed subject, and the abstract. From the 51 papers remaining, we excluded 20 studies for not presenting full text nor accessible in the databases, 2 studies for not being in the selected languages (Chinese and Korean) for the investigation, and 7 studies for not complying with the research objectives. Hence, 22 composed the final sample of the review, as exposed in Figure 2.

Figure 3 demonstrates the characterization of studies, including country, year of publication, title of the article, journal, and study design.

**Figura 3 -:** Characteristics of the studies that integrated the sample of the scoping review sample, according to country/year of publication, article title, journal, study design. Lisbon, Portugal, 2023

Country/Year of publication	Title	Journal	Type of study
Australia 2022	Assessment of the accuracy of the CALCULATE scale for pressure injury in critically ill patients^(^ 26 )	Australian Critical Care	Prospective cohort
Thailand 2020	Comparison of four pressure ulcer risk assessment tools in critically ill patients^(^ 27 )	Nurse Critical Care	Descriptive and prospective
Sweden 2020	Development and validation of an ICU*-specific pressure injury risk assessment scale^(^ 28 )	Scandinavian Journal of Caring Sciences	Prospective
United Kingdom 2020	Meta-analysis: Predictive validity of Braden for pressure ulcers in critical care^(^ 29 )	Nurse Critical Care	Literature review with meta-analysis
Canada 2019	Prediction Model for Hospital-Acquired Pressure Ulcer Development: Retrospective Cohort Study^(^ 30 )	JMIR Medical Informatics	Retrospective cohort
Brazil 2018	Evaluation of the accuracy of the CALCULATE and Braden scales in the prediction of the risk of pressure injury in the intensive care unit^(^ 31 )	Master’s Thesis	Retrospective cohort and analytical
USA† 2017	Usefulness of the Braden Scale in Intensive Care Units - A Study Based on Electronic Health Record Data^(^ 32 )	Journal of Nursing Care Quality	Retrospective
Australia 2017	Predictive ability of the EVARUCI scale and COMHON index for pressure injury risk in critically ill patients: A diagnostic accuracy study^(^ 33 )	Australian Critical Care	Retrospective cohort
United Kingdom 2017	Predictive validity of the Braden scale for assessing risk of developing pressure ulcers and dependence-related lesions^(^ 34 )	Journal of Wound Care	Longitudinal and prospective
Netherlands 2017	Validity of the current risk assessment scale for pressure ulcers in intensive care (EVARUCI) and the Norton-MI scale in critically ill patients^(^ 35 )	Applied Nursing Research	Descriptive, prospective
USA† 2017	Predicting the Risk for Hospital-Acquired Pressure Ulcers in Critical Care Patients^(^ 36 )	Critical Care Nurse	Observational retrospective
Spain 2017	Predictive validity and reliability of the Braden scale for risk assessment of pressure ulcers in an intensive care unit^(^ 37 )	*Medicina Intensiva*	Analytical, observational, longitudinal, and prospective
United Kingdom 2015	Part 2: pressure ulcer assessment: implementation and revision of CALCULATE^(^ 38 )	Nurse Critical Care	Prospective
United Kingdom 2015	Part 1: Pressure ulcer assessment – the development of Critical Care Pressure Ulcer Assessment Tool made Easy (CALCULATE)^(^ 39 )	Nurse Critical Care	Literature review
Brazil 2015	Evaluation of the pressure ulcers risk scales with critically ill patients: a prospective cohort study^(^ 40 )	*Revista Latino-Americana de Enfermagem*	Prospective cohort
USA† 2015	Predictive validity and reliability of the Turkish version of the risk assessment pressure sore scale in intensive care patients: results of a prospective study^(^ 41 )	Ostomy Wound Management	Prospective
Spain 2015	Validation of EMINA and EVARUCI scales for assessing the risk of developing pressure ulcers in critical patients^(^ 42 )	*Enfermería Intensiva*	Observational, correlational and prospective
Czech Republic 2014	Validity of pressure ulcer risk assessment scales: Review^(^ 43 )	Central European Journal of Nursing and Midwifery	Literature review
South Korea 2013	Reusability of EMR^‡^ Data for Applying Cubbin and Jackson Pressure Ulcer Risk Assessment Scale in Critical Care Patients^(^ 44 )	Healthcare Informatics Research	Retrospective
Portugal 2013	Validation of two pressure ulcer risk assessment scales among chinese ICU* patients^(^ 45 )	*Revista de Enfermagem Referência*	Longitudinal and prospective
Brazil 2011	Accuracy of two pressure ulcer risk scales for patients within critical condition^(^ 46 )	*Revista Enfermagem*	Longitudinal and prospective
Australia 2009	Comparison of the predictive validity among pressure ulcer risk assessment scales for surgical ICU* patients^(^ 47 )	Australian Journal of Advanced Nursing	Non‑experimental prospective study

*ICU = Intensive Care Unit; †USA = United States of America; ^‡^EMR = Electronic Medical Records

The analysis of the selected studies allowed us to determine that through the first article included^(^ 47 ) in this scoping review, dated from 2009, it is noticeable the increasing concern about this theme because from 2015 there was an increase of 72% (=16) of published articles.

The results of the studies allow us to subdivide the instruments into two big categories: the generalists, which are applicable in all care contexts, and the specifics, which are directed at adults in critical situation hospitalized in ICU. Thus, we identified six generalist instruments: Braden scale^(^ 36 ^,^ 26 ^-^ 27 ^,^ 29 ^-^ 32 ^,^ 34 ^,^ 37 ^,^ 40 ^,^ 43 ^-^ 47 ), Braden scale [ALB(Albumin)]^(^ 27 ), Emina^(^ 42 ), Norton MI [Modified by INSALUD (*Instituto Nacional de Salud Espanhol*)]^(^ 35 ), RAPS (Risk Assessment Pressure Sore)^(^ 41 ) and Waterlow^(^ 40 ^,^ 46 ), and seven specific instruments: CALCULATE (Critical Care Pressure Ulcer Assessment Tool made Easy)^(^ 26 ^-^ 27 ^,^ 31 ^,^ 38 ^-^ 39 ), COMHON index (Nutrition and Hemodynamic Oxygenation of Conscious Mobility)^(^ 27 ^,^ 34 ), Cubbin & Jackson scale^(^ 44 ^-^ 45 ^,^ 47 ), EVARUCI (*Escala de Valoración Actual del riesgo de desarrollar Úlceras por presión en Cuidados Intensivos*)^(^ 33 ^,^ 35 ^,^ 42 ^-^ 43 ), RAPS-ICU (Intensive Care Units)^(^ 28 ), Song & Choi^(^ 47 ) and Suriaidi and Sanada^(^ 43 ).

The Braden scale composes six subscales: sensory perception; moisture; activity; mobility; nutrition, and friction/shear. The user selects a score that varies from one to four in the subscales, except the subscale friction and shear, which scores from one to three, obtaining a total score from six to twenty-three points, the lower the result, the higher the risk of developing PU^(^ 36 ^,^ 26 ^-^ 27 ^,^ 29 ^-^ 32 ^,^ 34 ^,^ 37 ^,^ 40 ^,^ 43 ^-^ 47 ). The Braden scale (ALB)^(^ 27 ) is a modified version of the Braden scale, in which the nutritional subscale is based on serum albumin (serum albumin 35 g/L = 4). The other factors are evaluated in the same way as in the original Braden scale.

Norton MI scale is a generic scale applicable in different contexts that contemplates five parameters: mental condition, mobility, activity, physical condition, and incontinence, scored from 1 to 4 to obtain a total score from 5 (maximum risk) to 20 (minimal risk). This scale considers the classification of risk as follows: 5 to 11 is considered very high risk, 12 to 14 is moderate risk, and > 14 is minimum or no risk^(^ 35 ). The Emina scale derives from Norton and contains five risk factors: mental condition, mobility, incontinence, nutrition, and activity, scored from 0 to 3 in each one of the subscales, in which the higher the score, the higher the risk of developing PU^(^ 42 ).

The RAPS scale is composed of 12 variables based on the risk factors of the Norton, modified Norton and Braden scale: general physical condition, activity, mobility, food intake, fluid intake, moisture, sensory perception, friction and shear, skin condition, body condition, body temperature, and serum albumin values. The lowest scores indicate a higher risk of developing PU^(^ 43 ). Developed and validated through RAPS, the RAPS-ICU scale assembles three items: vital organ failure, mobility, moisture, sensorial perception, consciousness level, and special treatment, under mechanical ventilation, continuous dialysis and/or inotropic drugs. The score is from 1 to 4, except for vital organ failure, which is scored from 1 to 3, obtaining possible punctuation that varies from 6 to 23, in which the lowest score indicates a higher risk for developing PU^(^ 26 ).

The Waterlow scale assesses seven main topics: height/weight relationship, skin evaluation in risk areas, age/gender, continence, mobility, appetite, and medication. It also composes four items that score specific risk factors: tissue malnutrition, neurological deficit, surgery time superior to two hours, and trauma under the lumbar spine. The higher the score, the higher the risk of developing PU^(^ 40 ^,^ 46 ).

The CALCULATE, in its original version, is composed of eight risk factors, each one receives a point, and the total score is utilized to foresee the risk of PU, which may vary between 0 to 8. The higher the result, the higher the risk of developing PU^(^ 26 ^-^ 27 ^,^ 31 ^,^ 38 ).

The COMHON index includes risk factors inherent in an ICU, composed of five items: conscious level, mobility, hemodynamics state, oxygenation, and nutrition, scored from 1 to 4. The scores of cut-off proposed for this index are 5-8 points = low risk; 9-13 points = moderate risk; and 14-20 = high risk^(^ 27 ^,^ 33 ).

The Cubbin & Jackson scale consists of ten specific risk factors: age, weight, general skin condition, mental state, mobility, hemodynamics state, oxygen requirement, nutrition, incontinence, and hygiene. Each item has a scale of 4 points; hence, the maximum score is 40. The higher the score, the higher risk of developing PU^(^ 44 ^-^ 45 ^,^ 47 ).

The EVARUCI assesses four parameters: conscious level, hemodynamics, respiratory state, and mobility of the patient, each one of these parameters scores from 1 to 4. A fifth category, called “other,” evaluates the risk factors such as temperature, skin condition, blood pressure, patient’s prone position, and time of stay in the ICU. The total score varies from 4 - minimal risk - to 23 - maximum risk^(^ 33 ^,^ 35 ^,^ 42 ^-^ 43 ).

The Song & Choi is composed of six subscales from the Braden scale and two additional subscales: body temperature and amount of medication (analgesics, sedatives, and anticoagulants). Each subscale is evaluated from 1 to 3 or 4, and the scores vary from 8 to 31. The lowest indicate a higher risk of developing PU^(^ 47 ).

The Suriaidi and Sanada was developed in Indonesia, especially for intensive care, and is composed of three subscales: interface pressure scored from 0 to 3, body temperature scores from 0 to 4, and cigarette smoking scored from 0 to 2. The total score oscillates from 0 to 9, and the maximum value indicates a superior risk of developing PU^(^ 43 ^,^ 48 ).

We organized the results regarding the second and third objectives of this scoping review in Figures 4 (instrument performance indicators) and 5 (users’ appreciation regarding the use/limitations of the instruments), respectively.

**Figura 4 -:** Instruments performance indicators. Lisbon, Portugal, 2023

Mapped instruments	Instruments performance indicators
**Braden scale**	S* 66.5%; S† 62.2%; PPV^‡^ 12.5%; NPV^§^ 98.5%; AUC^||^ 0.69^(^ 30 ). AUC^||^ 0.61^(^ 31 ). S* 81%; S† 56%, PPV^‡^ 65%; NPV^§^ 74%; AUC^||^ 0.70^(^ 32 ). S* 90%; S† 26%; PPV^‡^ 31%; NPV^§^ 78%; AUC^||^ 0.63^(^ 34 ). S* 74.4%; S† 78.6; PPV^‡^ 28.6; NPV^§^ 96.4; AUC^||^ 0.79^(^ 36 ). S* 66.7%; S† 55.8%; PPV^‡^ 11.7%; NPV^§^ 95%; AUC^||^ 0.66^(^ 37 ). S* 41%; S† 21%; AUC^||^ 0.29^(^ 40 ). S* 78%, 95%, 71.4%; S† 29%, 45%, 83.1%; PPV^‡^ 70%, 52%, 31.3%; NPV^§^ 38%, 94%, 96.4%^(^ 42 ). S* 93.2%; S† 16.6%; PPV^‡^ 15.6%; NPV^§^ 93.7%; AUC^||^ 0.71^(^ 44 ). S* 91.7%; S† 63.0%; PPV^‡^ 19.0%; NPV^§^ 98.8%; AUC^||^ 0.15^(^ 45 ). S* 31.2%; S† 88.2%; PPV^‡^ 71.4%; VPN^§^ 66.4%^(^ 46 ). S* 92.5%; S† 69.8%, PPV^‡^ 40.6%, NPV^§^ 97.6%; AUC^||^ 0.88^(^ 47 ). AUC^||^ 0.71; 0.70^(^ 27 ). AUC^||^ 0.67^(^ 27 ). S* 89%; S† 28%; AUC^||^ 0.78^(^ 29 ).
**Braden (ALB) scale**	AUC^||^ 0.74^(^ 27 ).
**Emina scale**	S* 94.3%; S† 33.3%; PPV^‡^ 35.7; NPV^§^ 93.7; AUC^||^ 0.63^(^ 42 ).
**Norton MI scale**	S* 94.05%; S† 40.47%; PPV^‡^ 26.22%; NPV^§^ 96.80%; AUC^||^ 0.77%^(^ 35 ).
**RAPS**	S* 74.2%; S† 31.8%; PPV^‡^ 38.7%; NPV^§^ 91.3%; AUC^||^ 0.5^(^ 41 ).
**Waterlow scale**	S* 71%; S† 47%; AUC^||^ 0.57^(^ 40 ). S* 100%; S† 11.7%; PPV^‡^ 100%; NPV^§^ 100%^(^ 46 ).
**CALCULATE**	AUC^||^ 0.74^(^ 31 ). AUC^||^ 0.71^(^ 27 ). AUC^||^ 0.91;0.92^(^ 26 ).
**COMHON index**	S* 82.8%; S† 51.5%; PPV^‡^ 55.2%; NPV^§^ 80.6%; AUC^||^ 0.7^(^ 34 ). AUC^||^ 0.61^(^ 27 ).
**Cubbin & Jackson scale**	S* 72.0%; S† 68.8%; PPV^‡^ 27.7%; NPV^§^ 93.7%; AUC^||^ 0.76^(^ 44 ). S* 33.3%; S† 95.3%, PPV^‡^ 40.0%, NPV^§^ 93.8%; AUC^||^ 0.09^(^ 45 ). S* 95%; S† 81.5%, PPV^‡^ 53.5%, NPV^§^ 98.6%; AUC^||^ 0.90^(^ 47 ).
**EVARUCI**	S* 80.2%; S† 69.1%; PPV^‡^ 48.3%; NPV^§^ 90.7%; AUC^||^ 0.82^(^ 33 ). S* de 80.4%; S† 64.4%; PPV^‡^ 33.7%; NPV^§^ 93.6%; AUC^||^ 0.75^(^ 35 ). S* 92.4%; S† 42.9%; PPV^‡^ 38.8%; NPV^§^ 93.5%; AUC^||^ 0.67^(^ 42 ). S* 100%; S† 68.6%; PPV^‡^ 40.7%; NPV^§^ 10%; AUC^||^ 0.93^(^ 43 ).
**RAPS ICU**	S* 88%; E† 37%; AUC^||^ 0.71^(^ 28 ).
**Song & Choi scale**	S* 95%; S† 69.2%, PPV^‡^ 40.8%, NPV^§^ 98.4%; AUC^||^ 0.89^§(49)^.
**Suriaidi and Sanada scale**	S* 28.4%; S† 81%; PPV^‡^ 83%; NPV^§^ 65%; AUC^||^ 0.88^(^ 43 ).

*S = Sensitivity; †S = Specificity; ^‡^PPV = Positive Predictive Value; ^§^NPV = Negative Predictive Value; ^||^AUC = Area Under the Curve

**Figura 5 -:** Users’ appreciation of the instruments. Lisbon, Portugal, 2023

Mapped instruments	Users’ appreciation regarding the use/limitations of the instruments
**Braden scale**	“Further development and modification of this tool or generation of a new tool with higher predictive power is warranted”^(^ 29 ). “It is limited in predicting pressure ulcer risk factors”; “It requires that additional elements be applied to assess pressure ulcer risk in patients in ICU*”; “We found relatively low predictability of the tool”; “More research should be carried out to enhance the validity of the tool”^(^ 32 ). “The risk for hospital-acquired pressure ulcers was overpredicted”^(^ 36 ). “The risk of developing pressure ulcers is overestimated, and hence, it is difficult to conclude anything about the predictive capacity of this scale”^(^ 37 ). “The Braden scale was shown to be a good screening instrument”^(^ 40 ).
**Braden (ALB) scale**	“Based on AUC†, the Braden (ALB) scale demonstrated the best performance among risk assessment tools examined in this study, followed by CALCULATE, the Braden scale, and COMHON index”; “The standard laboratory indexes must be used as supplementary risk indicators of pressure ulcer”^(^ 27 ).
**Emina scale**	“At the usual cut-off point, it is less effective in the detection of the risk of pressure injuries in the critical patient”; “It classifies the great majority of patients as high risk”^(^ 42 ).
**Norton MI scale**	“It is an easy-to-use scale with clear definitions and criteria, which prevent variability between interobservers”; “One of the limitations is its simplicity, since it doesn’t include specific risk factors”; “More validation studies are necessary in the intensive care field”; “It may not be the most adequate scale to assess the risk in an ICU*, as it does not take into account specific risk factors”^(^ 35 ).
**RAPS**	“In this study, the RAPS scale was found to have acceptable reliability and poor validity […] to detect ICU* patients at risk of developing pressure ulcers”^(^ 41 ).
**Waterlow scale**	“Waterlow scale proved to have better predictive power than the Braden scale”^(^ 40 ). “Waterlow scale showed better scores and validity coefficients in risk assessment for pressure ulcers than the Braden scale”^(^ 46 ).
**CALCULATE**	“It presented better accuracy when compared to Braden scale”; “The transcultural translation was not carried out due to the objectivity of the scale and easy applicability”^(^ 31 ). “It was relatively simple to implement”; “We used an assessment scale from 1 to 5 (1 = hard and 5 = easy) to establish the facility to use the tool in practice. All nurses evaluated the tool as 3, 4, or 5, and the majority (65%) evaluated the tool with the highest score of 5 (easy)”; “less bureaucratic work”; “Easy to use and appropriate tool”^(^ 38 ). “It offers an important contribution towards the advancement and development of critical care pressure ulcer risk assessment”; “In the future, studies must concentrate their work to enhance risk factor validation and test the reliability and ponderation of each factor as a risk predictor”^(^ 39 ).
**COMHON index**	“It is easy to use”^(^ 33 ). “The COMHON index had a relatively unsatisfactory performance in this study. However, presented higher specificity among the tests studied”^(^ 26 ).
**Cubbin & Jackson scale**	“The Cubbin and Jackson scale had a slightly better performance than the Braden scale”^(^ 44 ). “Participants of the study found it difficult to apply in their practice”^(^ 45 ). “The Cubbin and Jackson scale was considered to be more reliable and valid than the Braden and Song Choi scale”^(^ 47 ).
**EVARUCI**	“Currently is the scale with the lower number of items, saving assessment time”^(^ 33 ). “It is an easy-to-use scale with clear definitions and criteria that avoid variability among observers. Besides, it includes an operational definition of the term”^(^ 35 ). “The EVARUCI Scale, specially developed for ICUs*, showed good values of the validity indicators”^(^ 43 ).
**RAPS ICU**	“ICU* staff perceived the RAPS-ICU as relevant and easy to use in clinical practice”; “The instrument can predict the development of pressure injuries during the stay at ICU* with good sensitivity and acceptable specificity. Hence, the scale could be used to identify patients in the ICU* with pressure injuries risk”; “It needs to be used and validated in future studies”^(^ 28 ).
**Song & Choi scale**	’It is one of the most known and favored scales in acute hospital environments in Korea”^(^ 47 ).
**Suriaidi and Sanada scale**	“The Suriaidi and Sanada scale, specially developed for ICUs*, showed good values of the validity indicators”^(^ 43 ).

*UCI = Intensive Care Unit; †AUC = Area Under the Curve

## Discussion

The recommendation of the Braden scale for adults in critical situation hospitalized in the ICU must be careful because it showed a high rate of false positives^(^ 29 ^-^ 30 ), which attributes to an overestimated risk for the PU prediction^(^ 36 ^,^ 37 ). Studies show that almost the totality of patients were classified as at risk, obtaining high sensibility and NPV and relatively low specificity and PPV values. That grants the Braden scale insufficient predictive validity and low precision in risk prediction. This observation is in agreement with previous studies^(^ 13 ^,^ 49 ) carried out that demonstrate the Braden scale is not a useful tool, that is, it does not have reliable applicability to the population in question, assisted in ICU. Thus, results can be achieved by implementing unnecessary and potentially costly preventive interventions. Another study, when compared to other generalist scales in a ward or intensive care unit, presents better results regarding its predictive value^(^ 50 ).

By the analysis of the included studies, we verified that the cutoff points vary from 12 to 16 and that the lower the cutoff point, the better the AUC values, which suggests that in ICU, the cutoff point must be inferior to 16^(^ 37 ). Other studies^(^ 40 ^,^ 45 ) presented disparate AUC values regarding the size of the participants’ sample. The interpretation of the results must be carried out with caution, not recommending its generalization.

Researchers that utilized the Braden scale suggest additional modifications to this tool, such as the inclusion of specific risk factors for adults in critical situation hospitalized in the ICU, once Braden’s subscales showed to be inadequate, being one of the reasons for the limitation in the PU evaluation in patients hospitalized in the ICU^(^ 29 ^,^ 32 ^,^ 51 ).

Due to relatively low inter-rater confidence values, a modification was carried out in the Braden scale in 2016, substituting the nutrition subscale with the serum albumin, originating the Braden (ALB) scale^(^ 52 ). The Braden (ALB) obtained a slightly inferior validity in comparison to the original Braden, with AUC values of 0.813 in comparison to 0.859 respectively; however, the inter-rater confidence increased significantly^(^ 52 ). Previous results counter the current data^(^ 27 ) when they affirm that the Braden (ALB) scale presents a superior AUC value to the CALCULATE scale, Braden, and COMHON index. More predictive validity studies are necessary on this scale to obtain more reliable and trustworthy results.

The Waterlow scale, developed through the Norton scale, has as base specific risk factors for ICU, and its utilization is considered complex, with moderate to good sensibility values but with low specificity values. These values confer to it a limited efficacy in the prediction of PU risk, proved by AUC values. For this reason, it is necessary to carry out more tests for this scale^(^ 44 ). In a recent study carried out in 2022^(^ 53 ) that compares the precision of the scales Braden and Waterlow for PU risk assessment in the ICU, the Waterlow scale obtained a slightly low predictive validity than Braden’s, disagreeing with the results of this research, which presented better predictive power, with better scores and validity coefficients^(^ 40 ^,^ 46 ).

The Emina scale is a tool developed and validated in Spain by nurses from the *Instituto Catalán de la Salud* to be utilized in hospital environments in services of short and long hospitalization, although it has not been validated for critical patients^(^ 42 ). In commonly used cutoff points, it was less efficient to detect the PU risk because it classifies the majority of critical patients as high risk. In the included study^(^ 42 ), the value of the cutoff point was increased from 4 (proposed by the original study of the scale) to 10, aiming at decreasing the number of false positives, considered one of the limitations of the scale. Hence, the Emina scale presents limitations for its use in the population which it was not validated for^(^ 42 ).

The Norton MI was adapted by INSALUD in 1996 through the original Norton scale, attending the validity and reliability criteria^(^ 54 ). Although more studies are necessary for the validation of this instrument in ICU, it will be able to be utilized for the risk assessment of PU^(^ 35 ). This scale is simple, it does not include specific risks for critical patients hospitalized in the ICU, which can be considered a limitation of its use^(^ 35 ).

The RAPS scale was developed for a Swedish population that speaks English and presents itself as the most commonly used scale in this country^(^ 28 ). When applied to adults in critical situation in the ICU, it presents low specificity values and PPV, just as the AUC values (0.5)^(^ 39 ), which demonstrates a low discriminatory capacity, not recommending its use. We did not find in the international literature other studies that evidenced its use in the ICU.

The EVARUCI was developed specifically for critically ill hospitalized in ICU^(^ 33 ) and includes specific risk factors for this population, considering its clinical lability^(^ 55 ). It is a validated scale, presenting adequate reliability and a very high inter-rater agreement^(^ 55 ). It presented good values of performance indicators, a general rule, originating a good predictive capacity of the instrument. The sensibility scores were slightly inferior to the ones obtained by other validated scales, such as Norton, Braden, Waterlow, and Song & Choi, but the specificity scores were very superior to the others^(^ 33 ^-^ 35 ). Regarding the AUC values, they oscillate between 0.67 to 0.93, which according to Marôco^(^ 17 ), presents a very good and acceptable discrimination, corresponding to a PU predictive capacity from moderate to exceptional. The users’ opinion is essentially positive recommending its use since it is easy to fill out and use^(^ 33 ^,^ 35 ).

The CALCULATE, developed as a specific instrument for critical patients, is the more recent scale for intensive care^(^ 38 ). A validation study concerning the Braden scale^(^ 30 ) demonstrated that the last one was more internally consistent; however, CALCULATE presented better reproductive accuracy (with superior AUC value). Thus, this instrument was considered repeatable and presented a better success rate in the prevention of PU. Nonetheless, presented limitations regarding the translation to Portuguese that were not adjusted/corrected due to easy usage and objectivity. This limitation can also be interpreted as positive. It is easy to use in clinical practice and recommended for patients hospitalized in the ICU^(^ 39 ).

More recently, a prospective cohort study compared the precision of the CALCULATE scale with the Braden’s in the prediction of PU risk in critical patients, and it concluded that CALCULATE can be more precise than the Braden scale as an instrument to evaluate the risk of developing PU in critical patients, presenting very promising AUC values of 0.91 and 0.92^(^ 26 ). CALCULATE can be a very easy and appropriate assessment instrument to assist in the precise identification of patients with a high risk to develop PU^(^ 39 ).

The Cubbin & Jackson scale was specifically projected for patients hospitalized in the ICU. When compared to the Braden^(^ 44 ) and Song & Choi^(^ 47 ) scale, it has a higher capacity to predict PU development in adults hospitalized in ICU. This scale has not been widely accepted due to the heterogeneity of the results related to AUC, being hard to obtain a reliable and correct conclusion regarding its predictive value^(^ 45 ^,^ 56 ).

The COMHON index was created as a result of a multicenter observational study to develop a specific scale with the objective of assessing the PU risk in adults hospitalized in the ICU^(^ 57 ). This validated scale may be a useful instrument to correctly classify critical patients of low risk. However, due to low specificity and NPV^(^ 34 ), the high-risk values obtained do not directly implicate the development of PU. In 2021 a prospective study^(^ 27 ) was developed that compared four PU risk assessment scales in critical patients, and the COMHON index obtained the worst result, with moderate values of AUC. Aiming at improving its performance, we suggest the modification of the nutritional subscale because serum albumin seems to be a more sensible predictor in the development of PU than via feeding^(^ 36 ^,^ 52 ).

The RAPS-ICU scale was developed and validated through the RAPS scale^(^ 28 ). We considered the instrument as easy to use and can predict the development of PU during the stay in the ICU. It presents acceptable sensibility and specificity values. It is a recent instrument that needs to be submitted to other studies for its evaluation.

The Song & Choi scale was developed and validated based on the theoretical foundation of the Braden scale and is one of the most commonly used in South Korea^(^ 58 ). A single study demonstrated that the scale^(^ 47 ) presents elevated values of AUC, which attributes to it a high validity in the prediction of PU risk. We did not identify in the international literature other studies that confirm or counter this evaluation, which limits the possibility to utilize this scale.

The Suriaidi and Sanada scale, despite their good predictive capacity, present limitations related to the usage of two devices: a pressure sensor multi-pad type and a thermometer. The multi-pad sensor may not be very adequate in other countries, especially outside of Asia, due to physical differences among populations.

The use of the risk assessment instrument is an important measure in the preventive process, constituting an effective mechanism to the decrease of PU prevalence among hospitalized patients, in particular the critical patients. The results of this study may contribute to the nurse determining more adequate instruments for use in the ICU. Choosing the more precise instrument contributes thus to better sustainability of health institutions. Regarding the patients’ benefits, we highlight a shorter hospital stay, shorter damage, and better quality of life.

As a limitation, we identify the possibility of carrying out more broad research in terms of languages and with no time limitations, which could offer more results. On the other hand, we highlight the methodological heterogeneity in the studies we found, which restricted the possibility of comparing the results.

Future research that evaluates the usage and efficiency of specific scales for the risk assessment of PU in patients in critical situation hospitalized in the ICU may offer an important contribution to a better validation of the instruments.

## Conclusion

This scoping review identified a variety of instruments in the international literature for the risk evaluation of PU in adults in critical situation hospitalized in the ICU, which are divided into two groups: generalist and specific scales. About the generalists, we identified Braden, Braden (ALB), Emina, Norton-MI, RAPS, and Waterlow scales. Regarding the specifics, we identified the CALCULATE, COMHON index, Cubbin & Jackson, EVARUCI, RAPS-ICU, Song & Choi, and Suriaidi and Sanada scales.

Regarding its predictive value and usage in ICU, we indicated specific scales once they presented better results related to its use and discriminatory power. According to the research carried out, we concluded that the specific instruments with better results, in terms of performance indicators, are the EVARUCI and the CALCULATE. Concerning the users’ appreciation in relation to their opinion/limitations of the instrument, we highlight in first place CALCULATE, in second place EVARUCI, and in third place RAPS-ICU.
